# Youth Suicide Prevention Programming among the Mississippi Band of Choctaw Indians: Effects of the Lifelines Student Curriculum

**DOI:** 10.3390/children11040488

**Published:** 2024-04-18

**Authors:** John P. Bartkowski, Katherine Klee, Xiaohe Xu

**Affiliations:** 1 Department of Sociology and Demography, University of Texas at San Antonio, San Antonio, TX 78249, USA; xiaohe.xu@utsa.edu; 2 Bartkowski & Associates Research Team, San Antonio, TX 78258, USA; kklee182@gmail.com

**Keywords:** Native American, indigenous, suicide prevention, children, elementary, middle school, mental health

## Abstract

Suicide continues to be a leading cause of mortality for young people. Given persistent intersecting forms of disadvantage, Native American adolescents are especially vulnerable to mental health adversities and other suicide risk factors. The Mississippi Band of Choctaw Indians (MBCI) implemented the Choctaw Youth Resilience Initiative (CYRI), a five-year SAMHSA-funded project that began in 2019. This study uses Choctaw student pre-test/post-test survey data to examine the effectiveness of the Hazelden Lifelines Suicide Prevention Training curriculum for youth. A lagged post-test design was used, whereby post-surveys were administered at least one month after program completion. Several intriguing results were observed. First, the lagged post-test model was subject to some pre-to-post attrition, although such attrition was comparable to a standard pre/post design. Second, analyses of completed surveys using means indicated various beneficial effects associated with the Lifelines curriculum implementation. The greatest benefit of the program was a significant change in student perceptions concerning school readiness in response to a suicidal event. Some opportunities for program improvement were also observed. Our study sheds new light on suicide prevention training programs that can be adapted according to Native American youth culture. Program implementation and evaluation implications are discussed in light of these findings.

## 1. Introduction

Suicide in the United States has proven to be a widespread challenge, with more pronounced vulnerabilities evident according to age, race/ethnicity, and gender. In 2020, 66,017 violence-related deaths occurred in 48 U.S. states and territories, with the majority (58.4%) being suicides [1]. The increase in completed suicides has been especially alarming in youths aged 10–14, with the rate of suicides tripling from 2007 to 2018 [2,3]. Native American groups are at a heightened disadvantage for mental health crises, with compounding social and economic factors leading to an increased risk of adverse childhood experiences (ACEs) and limited resources to address mental health and environmental safety [4]. Native American youth, especially those aged 15–24, have a suicide rate 1.5 times higher than the general population, while also facing an increased risk for substance use and depression [5]. Research has shown that suicide attempts without suicidal ideation have become increasingly common, more so in American Indian samples than in the general population [6]. Common suicide-related risk factors for Native American youth include school or online bullying, domestic or physical abuse, lack of mental health resources, and limited trusted adults, among others [7]. Suicide prevention efforts among indigenous populations have largely produced lackluster results due to uncoordinated individual-based approaches [8]. However, culturally focused community interventions have generated positive results, as with the adaptable Question-Persuade-Refer program and other gatekeeper training curricula [8,9].

New research has underscored the importance of incorporating culturally based contexts into suicide prevention curricula. The results of a scoping review have demonstrated how centering indigenous knowledge and approaches within suicide prevention programs positively contribute to suicide prevention and intervention outcomes. Initiatives built upon comprehensive community engagement processes, which include indigenous culture and knowledge, have shown to substantially impact suicide-related outcomes at the individual and community levels [8,9]. In a similar review, nine interventions were mapped as research-supported methods for American Indian mental health and crisis support. The interventions fell into four main categories: school-based services, cultural adaptations, culture as treatment, and community involvement [5]. Results of culturally specific mental health interventions often demonstrate strength in suicide prevention knowledge and practice retention for American Indian youth. However, some studies have suggested that suicide prevention programming has a limited impact on the help-seeking behaviors of youth in distress [10]. Similarly, programs designed to facilitate help-seeking orientations have shown positive results; however, the degree and magnitude of impact have varied across programs. Evidence-based programs, such as Question-Persuade-Refer (QPR) and Hazelden Lifelines, often have the strongest outcomes in prevention and intervention measures for adults [11,12,13,14]. However, QPR suicide prevention programming is often focused on training adults who work with youth, rather than training children and adolescents themselves.

The specific locale in which programs are applied can also influence suicide prevention programming effectiveness. As with indigenous cultural integration, tailoring content to fit urban or rural settings is of key importance. Rural schools must overcome certain barriers that urban schools do not encounter, such as emergency or law enforcement distance and response times. Similarly, the availability of mental health professionals is largely limited or non-existent in certain rural locations [15]. For rural middle school students, factors such as gender (female), perceptions of school safety vulnerability, sensation seeking, psychological distress, bullying victimization, early initiation of drug use, and community disorganization often predict past-year suicidal ideation [16,17]. In a sample of two hundred and seventy-seven rural high school teachers, 86% reported that a student from their school had died by suicide within the last five years, with 65% believing that more than one student had died by suicide, and 70% being currently aware of students experiencing suicidality in their class [18]. Receiving professional development training on suicide and obtaining help from mental health clinicians predicted perceived self-efficacy in the identification of student suicide ideation and attempts. School teachers perceive that common barriers to helping students at risk of suicide include insufficient numbers of school-based mental health professionals and gaps in community mental health services. Indigenous youth often face intersecting factors that increase their risk of suicide. First, a rural location often fosters a sense of isolation, while also limiting health and emergency resources for the general population. Second, tribal communities often have limited mental health resources, in addition to increased poverty and childhood trauma [15]. In a 2014 series of Community of Practice webinars, the Suicide Prevention Resource Center (SPRC) developed recommendations for rural suicide prevention, which included training primary care professionals to screen for suicide risk and prioritize suicide prevention programming based on community or population needs by collaborating with local partners [19].

Our current study features evaluation results of Hazelden Lifelines Suicide Prevention Training for students in primary and secondary educational institutions run by the Mississippi Band of Choctaw Indians (MBCI). The curriculum is described as follows on the Hazelden website (https://www.hazelden.org/store/item/503138, accessed on 3 March 2024), from which it is available for purchase:

Hazelden Lifelines^®^ serves the whole community by providing suicide awareness resources for school administrators, faculty and staff members, parents, and students. Information about suicide and the role of students in suicide prevention is presented in easy-to-follow lessons. Lifelines Prevention^®^: Building Knowledge and Skills to Prevent Suicide is a comprehensive, whole-school suicide prevention curriculum that educates school faculty, parents, and students on the facts about suicide and their respective roles as suicide “preventers.” This newly revised edition uses updated language to reflect today’s best practices and youth culture … [along with] two videos and handouts explaining suicide warning signs and how to do a warm handoff when you suspect a student may need more care. In Lifelines^®^ Prevention, students participate in role-playing exercises that teach them what to do when faced with a suicidal peer. The exercises feature an emphasis on seeking adult help and frank discussions on the warning signs of suicide. In the process of teaching students how to help a friend, students who may be suicidal themselves will learn the importance of getting help as well. This compelling program is an ideal component to your community’s prevention programming.

There have been no studies, to our knowledge, that analyze the racial/ethnic correlations of school-based suicide prevention training programs among Choctaw youth. Further, published research results of Hazelden Lifelines curricula are limited in exploring the effectiveness of program implementation among youth. Pre-test/post-test evaluations have been used with great success across several evidence-based suicide prevention programs. However, our study expands on current research by incorporating a lagged post-test design with in-person and online MBCI training assessments completed by students during elementary and middle school hours. A lagged post-test design minimizes intrusion into the instructional delivery of program content, reduces evaluation costs and the prospect of over-testing, and has the potential to reveal longer-term effects of program participation than a conventional post-test methodology. This lagged design is also sufficiently flexible to respect the authority of school officials in terms of both program implementation and survey administration. Further, our approach combines both indigenous (cultural examples and contexts) and Western (Hazelden Lifelines national curriculum) approaches, with a general focus on encouraging youth to address mental wellness among their peers. Our study examines pre-test and lagged post-test program outcomes that cover school-specific suicide prevention awareness, age-appropriate suicide prevention knowledge and language, and self-rated efficacy in following school-based suicide prevention protocols. Consistent with typical program evaluation studies, we tested one overarching hypothesis. We expected to observe salutary changes in suicide prevention awareness and knowledge, which should be evident from pre-test to post-test. As a corollary to this general hypothesis, we further anticipated that changes in general suicide prevention awareness would be more robust than changes in specific suicide prevention knowledge from pre-test to post-test. This corollary expectation was due, in part, to our use of a lagged post-test design. Specific knowledge that may be quite evident immediately upon program completion is likely to wane somewhat over time, thereby dampening knowledge retention on a lagged post-test. We remained open to prospective results to the contrary, because Native American populations have been understudied with respect to suicide prevention training initiatives.

## 2. Materials and Methods

### 2.1. Research Design

A pre-test and lagged post-test survey design was used to assess student participant knowledge and perception changes before and after the Hazelden Lifelines suicide prevention training program for students was completed. This lagged approach was employed to determine if effects persisted after program participation, thus raising the effectiveness bar when compared with a standard post-test methodology administered immediately upon program completion. This approach also maximized training exposure time and minimized the diversion of academic time for the completion of program evaluation surveys. Pre-test (pre-training survey) and lagged post-test (post-training survey intentionally delayed by one to three months) responses from semesterly Lifelines training sessions (approximately 3 months) conducted between September 2022 and February 2024 were analyzed for this study. The student surveys were part of a programmatic quality improvement initiative (evaluation) and were not created with the intent to create generalizable knowledge that qualifies as research under the U.S. Office of Human Research Protections standards. Instead, the surveys and survey results were a baseline example for future replication, systematic investigation, and contributions to the further development of suicide prevention training. Due to the nature of this study as a program evaluation rather than formal research, standard IRB oversight did not apply. Nevertheless, all participating students were informed of the purpose of the pre-training and post-training surveys and the voluntary nature of their participation in this project. Informed consent was secured at the time of survey completion, which allowed for anonymous survey responses to be shared with the project evaluators. In line with ethical standards of survey practice, there is no way to trace survey responses to individual students. Due to this being a school-based program, parental consent was not shared with evaluators, and was instead provided by school leadership. The evaluators took all necessary steps to preserve data confidentiality and attend to related considerations.

The Lifelines suicide prevention training program for students was conducted as part of the Mississippi Band of Choctaw Indians’ (MBCI) Choctaw Youth Resilience Initiative—Mississippi (CYRI-MS), in partnership with the Mississippi Public Health Institute, and was funded by the Substance Abuse and Mental Health Services Administration (SAMHSA). The MBCI is a self-governing Native American tribe of 11,000 individuals historically facing cultural marginalization in a consistently impoverished, mostly rural, and racially segregated state. The MBCI is also the only federally recognized tribe in the state of Mississippi [20]. During 2001–2015, suicide rates were consistently higher in rural areas than in metropolitan areas for both males and females, with non-Hispanic American Indians/Alaska Natives having the highest rates of suicide in rural counties [21,22]. The CYRI project prioritized increasing the number of youth and youth-serving organizations able to identify and work with children and adolescents at risk of suicide. CYRI also aimed to increase the capacity of clinical service providers to assess and treat youth at risk of suicide.

CYRI supported the implementation of several suicide prevention strategies in the Choctaw tribal community, with the Hazelden Lifelines series of training programs (suicide prevention, intervention, and postvention) serving as a focal point for community-based suicide awareness and preparedness. In the CYRI program, however, only the Lifelines Prevention training was administered to students in partnered MBCI elementary and middle schools. As such, this study utilized the available prevention training pre-test and lagged post-test data. Hazelden Lifelines was previously included in the National Registry of Evidence-based Programs and Practices (NREPP) [23], but NREPP is no longer considered a valid determinant of evidence-based status. As one of the first school-based suicide prevention programs in the country, Lifelines has been adapted to reflect both program evaluation and increased knowledge about youth attitudes toward seeking help [23].

The Hazelden Lifelines Suicide Prevention student curriculum was implemented in participating MBCI elementary and middle schools. The curriculum was presented to students during class periods for one month during the fall or spring semesters. Class period training times were left to the ultimate authority of school leadership. All faculty selected by the school leadership to oversee the student Lifelines curriculum were trained by Hazelden Lifelines associates before the start of student training implementation. Faculty training included all topics covered by the student curriculum. A survey methodology was minimally discussed with faculty trainees. However, students were consented by overseeing faculty prior to the start of pre-training and post-training surveys. Consenting statements included mention that survey completion was anonymous and voluntary, and choosing not to complete surveys would not affect program involvement in any way. This study analyzed student trainees’ survey responses only and did not include faculty training surveys.

A pre-post survey design was developed by Hazelden Lifelines trainers in coordination with the CYRI evaluators. The evaluation tools were created to evaluate participating students’ self-rated knowledge and perceptions of suicide prevention topics in comparative analyses of before and after responses. Evaluation survey items were produced using previously validated questions and terminology from prior Lifelines-related training instruments, along with minor modifications to account for current training content. All items were carefully assessed to ensure maximum suitability for the age range to which they would be delivered (10–14-year-old students). The Lifelines pre-training and post-training surveys included a range of socio-demographic and knowledge rating items. The completion of the pre-/lagged-post evaluation surveys was anonymous, thereby making pre-/lagged-post matching unfeasible. CYRI supported the Lifelines training of MBCI school faculty and staff to implement Lifelines suicide prevention training materials across MBCI primary and secondary educational institutions. Suicide prevention specialists were certified to train youth by Hazelden Lifelines staff. However, to respect the authority and scheduling preferences of educational leaders, school officials oversaw the distribution of pre-test and lagged post-test evaluations. Several states have passed stringent suicide prevention laws to address gaps in resources for communities and schools. Principals in states with the most stringent laws reported having the most comprehensive suicide prevention programs [24]. In 2017, Mississippi passed a law that not only addressed school bullying that may lead to youth suicides, but also required all school district employees to have a minimum of two hours of suicide prevention training annually [25]. CYRI has aided in this prevention initiative through comprehensive training for students and faculty alike.

### 2.2. Data Collection and Sample

Participants included in this study were 5th–8th grade students who completed in-person Lifelines suicide prevention training delivered in tribal schools during class periods over the course of a semester. Pre-training and post-training survey responses from faculty-led training sessions conducted between 2022 and 2024 were analyzed for this study. All partner schools consented to the use of the Hazelden Lifelines program and the surveying of participating students for evaluation purposes. Prior to pre-test and post-test survey administration, the students were informed of the voluntary nature of the survey activity, and that would not impact their participation in the program. The pre-training survey consisted of four sociodemographic items (grade level, gender, race/ethnicity, and school name), and eleven knowledge and skill items about suicide. The post-training survey consisted of the same sociodemographic, knowledge, and skill items as featured in the pre-training survey, with one additional training usefulness item. The post-only item gauging usefulness is not analyzed in this study, but results are available by request. In total, 678 surveys were submitted to project evaluators from CYRI project leadership, of which the demographics are presented in Table 1. However, twelve pre-tests and nine post-tests were excluded from the data analysis presented in Table 2 due to incomplete survey responses or unparalleled data.

### 2.3. Statistical Analysis

Statistical tests were conducted consistent with standard scientific practice for analyzing ordinal data featuring Likert response options ranging from strongly agree to strongly disagree. Given the use of a five-point Likert scale with a neutral midpoint, the following coding scheme was employed: 5 = Strongly agree; 4 = Agree; 3 = Neither disagree nor agree; 2 = Disagree; 1 = Strongly disagree. No items were recoded, so the scale direction (higher scores indicate greater levels of agreement) was consistent across all survey items. Because the training program is designed to foster prevention-oriented knowledge and skills, survey items marked as positive attributes are survey prompts consistent with prevention beliefs and practices as taught in the Lifelines training. These positive (desirable) attributes were expected to increase from pre-test to post-test. At the same time, we anticipated greater pre-to-post disagreement with negative (undesirable) attributes, that is, incorrect statements that are at odds with the knowledge and skills taught through Lifelines. Given the anticipation that knowledge and skills consistent with the Lifelines curriculum should be enhanced from pre-test to post-test (increased agreement with positive attributes, decreased agreement with negative attributes), we employed one-tailed *t*-tests to compare means across each survey wave. These means were unadjusted but were similar to adjusted means after controlling for sociodemographic characteristics including grade level, gender, and race/ethnicity. We report these unadjusted means as well as the *t*-test results with *p*-values (significance levels) in Table 2. One-tailed tests were conducted to produce results, given expectations for an increase in positive attributes and a decrease in negative attributes over time. For each mean, we also calculated the 95% confidence interval, the results of which are also reported in Table 2. In program validation studies of this nature, it is not uncommon to report *p*-values that approach statistical significance (*p* < 0.10). This level of detail is helpful for the possible refinement of programmatic content, teaching emphasis, and survey indicators.

## 3. Results

The following results displayed in Table 1 were taken from the total pre (*n* = 367) and post (*n* = 311) surveys. Just over half of the participating students identified as female (pre *n* = 196, 53.4%; post *n* = 156, 50.2%) at the time of pre-test and post-test completion, respectively, with fellow participants identifying as male (pre *n* = 163, 44.4%; post *n* = 149, 47.9%). Most participants were Native American (pre *n* = 267, 72.8%; post *n* = 239, 76.8%), with a smaller amount identifying as multiracial (pre *n* = 100, 27.2%; post *n* = 72, 23.2%). The participating students included 5th grade (pre *n* = 75, 20.4%; post *n* = 76, 24.4%), 6th grade (pre *n* = 45, 12.3%; post *n* = 57, 18.3%), 7th grade (pre *n* = 93, 25.3%; post *n* = 71, 22.8%), and 8th grade (pre *n* = 154, 42.0%; post *n* = 107, 34.4%) youth from MBCI schools.

Table 2 displays the results of the survey data analyses. The left-most column features the item verbatim as presented in the survey. The next column to the right represents the pre-test mean (average) and 95% pre-test confidence interval based on the degree of agreement with each statement prompt, after which the number of valid responses is featured. Moving further to the right in Table 2, the post-test means and 95% confidence intervals are featured, along with the number of valid responses. Given the data limitations (relatively small sample size, no pre/post survey matching to preserve full anonymity) and our focus on evidence validation, we treated statistical significance (*p*-values) of *p* = 0.10 as borderline (approaching) significance, while recognizing that a threshold of *p* = 0.05 or less achieved actual statistical significance. The two right-most columns, respectively, indicate whether the change from pre-test to post-test reflected an increase in agreement (↑) or a decrease in agreement (↓). All survey items were analyzed with one consistent level of agreement scale in which 5 = Strongly agree. Therefore, distinctions could be drawn between survey items that represented a negative attribute and their positive attribute counterparts. Negative attribute survey questions consisted of items 1, 3, 4, 5, 8, and 11. A desirable change for these negative attribute items entailed a *decline* in agreement from pr-test to post-test. By contrast, items 2, 6, 7, 9, and 10 represented positive attributes, whereby an *increase* in agreement from pre-test to post-test reflected a desirable change. The items featured in Table 2 are verbatim as they appeared in the survey.

First, reviewing item one in Table 2, there was a notable decrease from pre-test to post-test mean outcomes when students were asked about young people being incapable of preventing suicide. The pre-to-post change in item one was therefore desirable. However, this change was not of a sufficient magnitude to achieve statistical significance. A similar pattern is observed for the next two items. Item two asked about having a trusted adult contact and exhibited an increase in mean agreement from pre to post, but did not achieve statistical significance for this positive attribute item. Item three, a negative attribute indicator about not breaking a suicidal friend’s confidence, decreased from pre to post. This change was desirable but failed to achieve statistical significance. In short, all three of these items operated in a desirable direction from pre to post, but the changes were not statistically significant and could therefore have been due to chance. Item four presented a troubling pattern. This item gauges the degree to which young people should solve their problems independently from adult involvement. Greater agreement at post-test was an undesirable trend, despite this item achieving statistical significance at the 0.07 level. Item five asked about avoidance of inquiring about suicidal thoughts with a friend. This item exhibited the smallest degree of change (slight increase) from pre to post, and was not statistically significant. Item six asked students to convey perceived suicide prevention preparedness in their school. There was a statistically significant increase (*p* = 0.034) in agreement with this statement from pre-test to post-test. These results indicated that students were more aware of school-based suicide prevention resources after the training than they were before it. Moreover, given the lagged post-test design used with the Lifelines curriculum, it is noteworthy that students retained this important point of knowledge well after the training had concluded.

Moving to item seven, students indicated that they would encourage someone displaying suicidal characteristics to seek help from a responsible adult. Although a slight increase was observed in this positive attribute, the pre-to-post mean comparison did not achieve statistical significance. Conversely, the next three items displayed either borderline or robust levels of statistical significance. Item eight inquired about students endeavoring to help a suicidal individual without adult intervention. This negative attribute showed a welcome pre-to-post decrease, and nearly achieved a 0.05 level of statistical significance (*p* = 0.060). Item nine asked students about perceived school readiness to intervene with a suicidal student and yielded robust statistical significance (*p* = 0.005) concerning the pre-to-post increase. This result, given the lagged post-test design, indicates that memorable discussions during training sessions were centered around school preparedness in suicide prevention. Item ten examined the degree to which students have an adult confidant. Item ten approached significance (*p* = 0.100), but fell short of the *p* < 0.05 threshold. The final question, item eleven, showed a slightly troubling but not statistically significant pre-to-post increase in not knowing how to respond to a friend experiencing suicidal ideation. In sum, approximately the same mean score was observed at pre-test and post-test.

In ancillary analyses, we sought to confirm the independent samples *t*-test results displayed in Table 2 using regression models. The results of the regression analyses are available upon request from the authors. The general pattern found in the independent samples *t*-tests featured in Table 2 was also present in the regression analyses, although the strength of statistical significance weakened slightly in the regression models.

**Table 2:** Pre/post changes in student suicide prevention knowledge and skills.

Item ^1^	Pre-Test Mean (95%CI)	*n*	Post-Test Mean (95%CI)	*n*	Change from Pre-Test	Statistical Significance ^2^
1. Young people can’t do very much to prevent youth suicide. (Negative)	2.75 (2.61–2.88)	351	2.67 (2.56–2.82)	301	↓ ^3^	0.178
2. It is important to have at least one adult you can talk to if something is bothering you. (Positive)	4.12 (4.01–4.23)	355	4.19 (4.05–4.28)	301	↑	0.169
3. A friend’s confidence in sharing suicidal feelings should never be broken. (Negative)	2.95 (2.82–3.08)	352	2.88 (2.74–3.02)	302	↓	0.224
4. Young people are at a point in their lives when they should not rely on adults for help with problems. (Negative)	3.05 (2.91–3.17)	353	3.20 (3.02–3.32)	298	↑	0.07
5. It is not a good idea to ask someone if they are thinking about suicide, because you may give them the idea to try it. (Negative)	3.17 (3.04–3.29)	351	3.18 (3.02–3.34)	300	↑	0.477
6. My school is prepared to help a student who might be thinking about killing themselves. (Positive)	3.68 (3.56–3.79)	349	3.84 (3.71–3.98)	299	↑	0.034
7. If a friend came to school in a bad mood and casually mentioned, “My family would be better off without me”, I would encourage that person to get help from a responsible adult. (Positive)	4.13 (4.04–4.22)	352	4.14 (4.04–4.24)	302	↑	0.413
8. I would try to help a suicidal friend without getting help from someone else. (Negative)	3.03 (2.90–3.16)	345	2.88 (2.73–3.01)	296	↓	0.060
9. I know what officials in my school will do if they learn about a student who is thinking about killing or hurting themselves. (Positive)	3.36 (3.20–3.49)	345	3.57 (3.46–3.72)	296	↑	0.005
10. There is at least one adult in my school that I could confide in about a concern of my own or about a friend’s concern. (Positive)	3.77 (3.65–3.87)	343	3.87 (3.73–3.98)	297	↑	0.100
11. If a friend told me that they were thinking about suicide, I would not know what to do. (Negative)	2.47 (2.35–2.62)	344	2.55 (2.41–2.67)	296	↑	0.186

^1^ 1 = Strongly disagree, 2 = Disagree, 3 = Neither agree nor disagree, 4 = Agree, 5 = Strongly agree. ^2^ One-tailed tests were conducted to produce these results given expectations for either an increase in positive attributes or a decrease in negative attributes over time. ^3^ Arrows (↑ ↓) denote increase or decrease changes in results from pre-test to post-test.

## 4. Discussion

Our current study analyzed the effectiveness of Hazelden Lifelines Suicide Prevention Training for Mississippi Band of Choctaw Indian (MBCI) tribal students. Suicide prevention trainings for primary and secondary education students (elementary through high school) have not been as widespread as prevention or gatekeeper trainings for adults [11,12,13,14]. However, programs that have been implemented are often most successful when using evidence-based curricula [9,10]. While Lifelines was once registered as an evidence-based program [23], it has not been formally evaluated using recent evidence-based standards. Our study tested one overarching hypothesis, namely that salutary changes in suicide prevention awareness and knowledge would be observed from pre-test to post-test. Our study also examined a secondary hypothesis, such that changes in general suicide prevention awareness would be more robust than changes in specific suicide prevention knowledge from program initiation to completion. The Lifelines program comprises three parts: prevention, intervention, and postvention. Only the prevention material administered to students was the subject of our study. Therefore, our study is exploratory and notable in its use of evaluation results from an under-studied suicide prevention training program administered to an underserved minority population [10]. A lagged post-test design that split the difference between a conventional post-test and a follow-up survey was employed for this study. Post-tests for this study were administered one to three months after the program concluded, thereby providing an opportunity for a methodological innovation to be piloted, given time constraints in partner schools. Additionally, the lagged post-test design of this study presented a unique methodology among the existing literature, in which traditional pre-to-post-test administrations are common. The results of our study expanded the fields of suicide prevention training among Native American youth and evaluation methodology.

Overall, analysis of pre-training and post-training surveys produced mixed results. Among the positive effects observed, most measures changed in a desirable direction from pre to post. The consistency of the results indicated moderate program effectiveness. However, statistical significance was not consistently observed. Among the statistically significant results that were observed, several centered around student perceptions of school readiness for suicidal crisis response. Specifically, item six (My school is prepared to help a student who might be thinking about killing themselves), item eight (I would try to help a suicidal friend without getting help from someone else), item nine (I know what officials in my school will do if they learn about a student who is thinking about killing or hurting themselves), and item ten (There is at least one adult in my school that I could confide in about a concern of my own or about a friend’s concern) yielded statistically significant results in desirable directions of agreement. This group of outcomes is a noteworthy achievement because, upon program completion, students appeared to have confidence in their school’s preparedness to react effectively were such a crisis to emerge. In theory, these results could also reflect students’ confidence in wider community support, as school officials and faculty are often key members of the tribe [26]. These findings are also consistent with relevant research detailing the successes of suicide prevention programs [5,7,11,27]. However, individual-level knowledge and skills were not consistently acquired through the curriculum. Similarly, results indicate somewhat of a persistent non-interventionist approach held by some students from pre-test to post-test completion that is contrary to Lifelines’ curricular content. Therefore, the Hazelden Lifelines Suicide Prevention student curriculum is moderately effective (as opposed to not effective or highly effective) among Native American youth in grades five through eight due, in large part, to the evaluation design of this study (lagged post-test). The Lifelines curriculum was most effective in raising general awareness about school-based suicide prevention.

We believe that the lack of statistically significant findings reported here was due primarily to the use of a lagged post-test design. The very modest increases or unexpected declines in individual-based knowledge and skills would likely be altered if surveys were administered immediately upon training completion. Knowledge and skills tend to be quite robust immediately after a program concludes and would probably have been detected on a conventional post-test administered during the final class period. These mixed results could also become more uniformly positive with a greater emphasis on individual contributions to suicide prevention within curricular content or when delivering this program. Therefore, we would recommend that a more conventional pre-test, post-test, and follow-up methodology be used to study the impacts of Lifelines’ programs going forward. It is worth noting that our evaluation efforts were tailored to school preferences, whereby school leadership had the final determination of when the program curriculum was delivered, when the pre-test and post-test surveys were administered, and precisely who oversaw program and survey completion. All faculty of partner schools were trained in prevention, intervention, and postvention techniques by Hazelden Lifelines staff. However, discussion of the survey protocol during training for school officials was somewhat limited. To determine the persistence of knowledge and skill gains, Lifelines recommends post-tests for students be delivered a minimum of one month following program completion [23]. While this approach is advantageous in that regard, a post-test administered at the end of the final training period would likely yield superior results, even if it is more cumbersome for schools. The value of the lagged post-test approach remains evident, as schools often critically consider diverting traditional instructional time to the surveying of students for special programming such as Lifelines. Therefore, a lagged post-test design is still advantageous compared with an absence of evaluation surveys, but perhaps should not replace a traditional end-of-program post-test.

The success of Hazelden Lifelines was transparent in results for school preparedness, student help-seeking from adults, confidence in school officials, and knowing a trusted adult in the school. However, a few limitations were present in our study. As mentioned, the lagged post-test design can increase the time allotted to teach the curriculum, but it can also hinder evaluating short-term knowledge gains and statistically significant pre-to-post-test comparisons. Additionally, as this study had an evaluation-based design, and schools had final authority in program implementation, our involvement in survey administration and curriculum dissemination was limited. While the use of cultural symbols and references was incorporated in training materials, the specifics were not shared with program evaluators and were, therefore, not directly measureable in this study. Research has highlighted the effectiveness of cultural inclusion in suicide prevention programs for Native American youth [5,6,7,8,26,28,29,30,31]. Future studies of Lifelines implementation would do well to investigate cultural adaptations in educational materials and their potential impact on knowledge gain and retention. Regarding the survey instrument, items assessing the establishment of protective factors for students could be expanded (i.e., cultural practices, community involvement and support) [26,32]. Going forward, future evaluation efforts would do well to consider applying survey protocol methods more directly within training materials and discussions for school faculty and staff that lead the implementation of student suicide prevention programming. Protocols could include participant protections (student anonymity), clarifying evaluation instruments as distinct from mental health assessments (not to be used for counseling recommendations), and best survey practices (students submit surveys anonymously, responses reviewed by evaluators only). Successful survey administration should also include online survey submissions, whereas our study utilized both online and hard-copy (printed PDF) survey responses. Technological limitations often lead to hard-copy survey use. However, the use of online surveys is preferred where feasible, as this method limits the possibility of participant identification and eases physical data collection burden.

## 5. Conclusions

Native American children are at higher risk of suicidal ideation and behaviors than any other minority ethnic group in the United States [26]. Suicide rates increased starkly from 2020 to 2021 for non-Hispanic American Indian and Alaska Native youth aged 10–24 [33]. The implementation of Hazelden Lifelines Prevention training for students offers a unique tailoring of suicide prevention curricula to indigenous youth ages 10–14. Our study evaluated the Lifelines suicide prevention training conducted in Mississippi Band of Choctaw Indians-affiliated primary and secondary educational institutions as part of the SAMHSA-funded Choctaw Youth Resilience Initiative between 2022 and 2024. Our results reveal the Hazelden Lifelines Prevention program for students to be highly effective in communicating school-based prevention efforts but, at most, moderately effective with regard to individual-level preparedness for suicidal crisis events. Where results were somewhat muted, they may be due to the use of a lagged post-test methodology rather than the immediate administration of a post-test survey. This study provides additional evidence of CYRI effectiveness because a previous study underscored the success of CYRI gatekeeper training [34]. Lifelines offers a unique opportunity to implement youth-focused suicide prevention programming in elementary, middle, and high schools. Efforts should be made to refine evaluation strategies. Further attention to expanding curricula in emphasizing individual efforts and practices during suicide events would potentially bolster program effectiveness for students.

## Figures and Tables

**Table 1:** Student sample characteristics.

Item	Pre-Test		Post-Test	
	*n*	Percentage	*n*	Percentage
Grade Level				
5th	75	20.4	76	24.4
6th	45	12.3	57	18.3
7th	93	25.3	71	22.8
8th	154	42.0	107	34.4
Total	367	100	311	100
Gender				
Female	196	53.4	156	50.2
Male	163	44.4	149	47.9
Other	8	2.2	6	1.9
Total	367	100	311	100
Race/ethnicity				
Native American	267	72.8	239	76.8
Multi-race	100	27.2	72	23.2
Total	367	100	311	100

## Data Availability

The data used to conduct this evaluation are proprietary and not suitable for public release due to grant funder restrictions and grant project data confidentiality terms. Please contact the lead author for more information about the data used to conduct this study.
